# Notes on the Newer Remedies, Their Therapeutic Application and Modes of Administration

**Published:** 1895-04

**Authors:** 


NOTES ON THE NEWER REMEDIES—THEIR THERAPEUTIC APPLICATION AND MODES OF ADMINISTRATION. By David Cerna, M. D., Ph. D., Demonstrator of Physiology and Lecturer on the History of Medicine in the Medical Department of the University of Texas; formerly Assistant in Physiology, Demonstrator of, and Lecturer on Experimental Therapeutics in the University of Pennsylvania, etc. Second Edition, Enlarged and revised. Philadelphia: W. B. Saunders, 925 Walnut Street. 1895. Price, $1.25.

The author does not claim this to be a work on therapeutics. Rather "Notes on the newer Remedies"—records of the therapeutic application of remedies which have multiplied so rapidly in the last five years—especially of those whose usefulness has been determined by clinical investigation.

A reference book of the kind which is much in advance of even our modern text-books, should be appreciated and welcomed by the profession, as it will enable them, with the smallest possible trouble, to keep up with this rapidly advancing branch of medical science.

The drugs are discussed in alphabetical order. The formula, the scientific and common name of each is given. Special attention is given to the therapeutic application and mode of administration. Both the Apothecaries' and metric systems are employed. We cannot too highly praise this little book, as it is simply invaluable, and fills a very vacant place in our library.

